# Both TALENs and CRISPR/Cas9 directly target the *HBB* IVS2–654 (C > T) mutation in β-thalassemia-derived iPSCs

**DOI:** 10.1038/srep12065

**Published:** 2015-07-09

**Authors:** Peng Xu, Ying Tong, Xiu-zhen Liu, Ting-ting Wang, Li Cheng, Bo-yu Wang, Xiang Lv, Yue Huang, De-pei Liu

**Affiliations:** 1 State Key Laboratory of Medical Molecular Biology, Institute of Basic Medical Sciences, Chinese Academy of Medical Sciences and Peking Union Medical College, Beijing, China; 2 Department of Biochemistry and Molecular Biology, Institute of Basic Medical Sciences, Chinese Academy of Medical Sciences and Peking Union Medical College, Beijing, China; 3 Department of Medical Genetics, Institute of Basic Medical Sciences, Chinese Academy of Medical Sciences, School of Basic Medicine Peking Union Medical College, Beijing, China

## Abstract

β-Thalassemia is one of the most common genetic blood diseases and is caused by either point mutations or deletions in the β-globin (*HBB*) gene. The generation of patient-specific induced pluripotent stem cells (iPSCs) and subsequent correction of the disease-causing mutations may be a potential therapeutic strategy for this disease. Due to the low efficiency of typical homologous recombination, endonucleases, including TALENs and CRISPR/Cas9, have been widely used to enhance the gene correction efficiency in patient-derived iPSCs. Here, we designed TALENs and CRISPR/Cas9 to directly target the intron2 mutation site IVS2-654 in the globin gene. We observed different frequencies of double-strand breaks (DSBs) at IVS2-654 loci using TALENs and CRISPR/Cas9, and TALENs mediated a higher homologous gene targeting efficiency compared to CRISPR/Cas9 when combined with the *piggyBac* transposon donor. In addition, more obvious off-target events were observed for CRISPR/Cas9 compared to TALENs. Finally, TALENs-corrected iPSC clones were selected for erythroblast differentiation using the OP9 co-culture system and detected relatively higher transcription of *HBB than the uncorrected cells*. This comparison of using TALENs or CRISPR/Cas9 to correct specific *HBB* mutations in patient-derived iPSCs will guide future applications of TALENs- or CRISPR/Cas9-based gene therapies in monogenic diseases.

β-Thalassemia is a monogenic blood disease characterized by reduced, abnormal or absent synthesis of β-globin chains^1^,^2^. It is estimated that 4.5% of the world wide population carry β-Thalassemia mutants^3^. And what’s more, this inherited disease, which is widely prevalent in Southern part of China and Southeast Asia, has threated millions of people’s lives for decades^4^. Individuals with β-thalassemia major, which was also known as Cooley’s anemia, is the most severe form of this disease, have ineffective erythropoiesis and hepatosplenomegaly^5^. β-Thalassemia is caused by more than 200 different point mutations and, rarely, deletions in the *HBB* gene^6^. Among the most frequent mutations are point mutations occurring in an intron, which can cause aberrant splicing. The IVS2-654(C > T) mutation is one common disease mutation of β-thalassemia in Southeast Asia^7^,^8^. The IVS2-654(C > T) mutation creates an aberrant 5’ splice site and activates a cryptic 3’ splice site within intron2 of the pre-mRNA, leading to the retention of nucleotides 580-652 of the second intron^9^. Thus, homozygous IVS2-654(C > T) mutations result in a deficiency of the correctly spliced β-globin transcript.

Hematopoietic stem cell (HSC) transplantation is an efficient means of curing β-thalassemia but is limited by the paucity of HLA-matched healthy donors^10^. Gene therapy in which a normal *HBB* gene is provided to a patient’s own HSCs via viral transduction is a potential treatment for β-thalassemia^11^,^12^. However, gene therapy using viral vectors that integrate randomly into multiple sites of the host genome may cause side effects. Patient-specific induced pluripotent stem cells (iPSCs) have recently been generated and hold great potential to cure monogenic diseases such as β-thalassemia^13^,^14^. However, standard homologous recombination by gene targeting in human pluripotent stem cells is relatively low, hampering their extensive application in cell therapy. Taken together, these drawbacks indicate the need for a more accurate and precise strategy for correcting mutations.

Recently, engineered nucleases, including zinc finger nucleases (ZFNs), transcription activator-like effector nucleases (TALENs) and clustered regularly interspaced short palindromic repeats (CRISPR)/CRISPR-associated 9(Cas9), have been widely used to generate double-strand DNA breaks (DSBs) to increase the efficiency of standard homologous recombination^15^,^16^,^17^,^18^. ZFNs have been used to target the *HBB* gene in α-thalassemia^19^. However, individual ZFN subunits influence the overall binding affinity of the reagent in a context-dependent manner, resulting in suboptimal gene targeting^20^. Compared to ZFNs, TALENs and CRISPR/Cas9 are easier to design and construct and have been favored by most investigators. TALENs and CRISPR/Cas9 were both recently reported to target the *HBB* gene in β-thalassemia^2^,^21^,^22^. However, the advantages and disadvantages of TALENs and CRISPR/Cas9 in targeting the *HBB* gene have not been fully investigated. Before their application in β-thalassemia gene therapy, the specificity and safety of both TALENs and CRISPR/Cas9 in targeting the *HBB* gene should be investigated.

In this study, we selected TALENs and CRISPR/Cas9 for the *HBB* intron2 IVS2-654 C > T mutation and observed efficient TALENs and CRISPR/Cas9 mediated homologous recombination respectively. CRISPR/Cas9 induced DSBs with greater efficiency than TALENs. For gene targeting near the IVS2-654 C > T mutation site, TALENs mediated higher homologous recombination efficiency than CRISPR/Cas9 in β-thalassemia-derived iPSCs. Moreover, hematopoietic differentiation of mutation-corrected iPSCs under the OP9 co-culture condition was induced and they showed relatively higher transcription of *HBB*, in contrast to the uncorrected parental cell lines.

## Results

### Specific TALENs and CRISPR/Cas9 targeting *HBB* IVS2-654 C > T mutation loci

**Figure 1:**
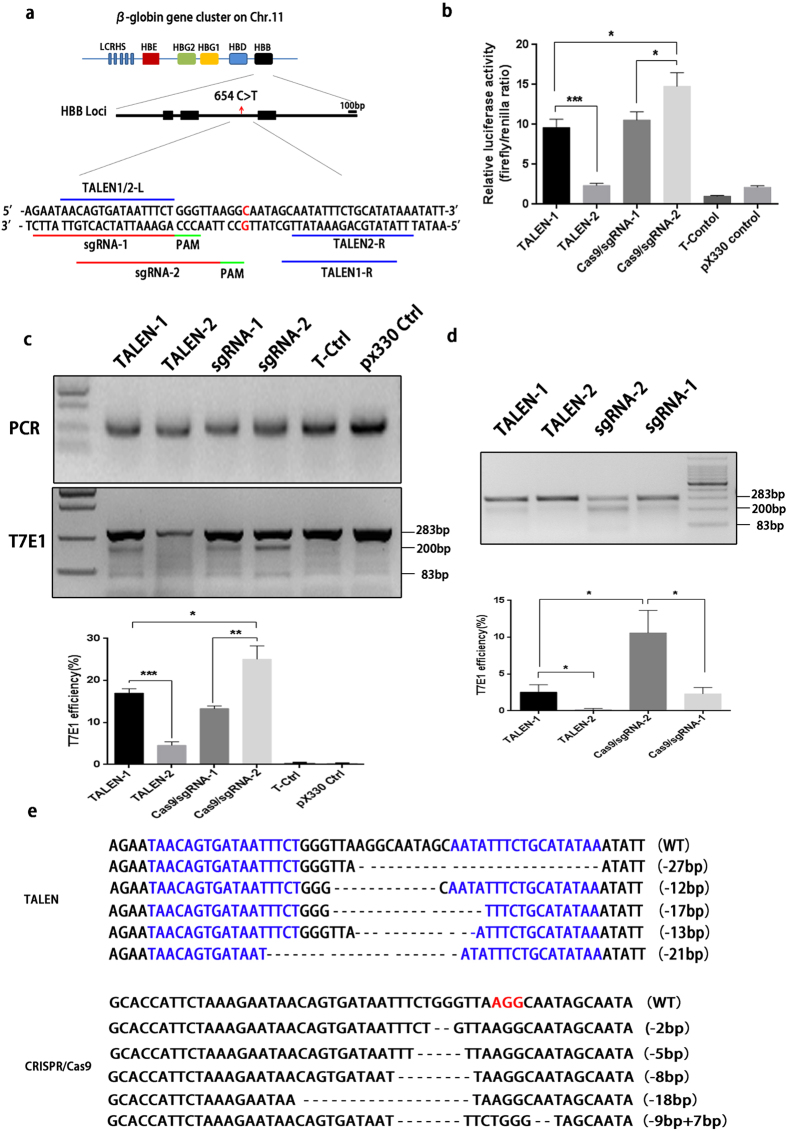
Both TALENs and CRISPR/Cas9 can directly and efficiently target the *HBB* gene IVS2-654 mutation site. (**a**) Two pairs of TALENs and two sites of CRSIPR guide RNA were designed for *in situ* targeting of the HBB gene IVS2-654 mutation site. TALEN sites are indicated by blue lines, and guide RNA sites are indicated by red lines. The red nucleotide in the middle sequence is the IVS2-654 mutation site. PAM: protospacer adjacent motif. (**b**) Evaluation of TALEN- and CRISPR/Cas9-mediated DNA cleavage by a SSA (single strand annealing) assay. HEK293 cells were separately co-transfected with one of the pairs of TALENs or one of the pairs of CRISPRs and pSSA-HBB-IVS2 and TK-Renilla. At 48 h after transfection, the ratio of firefly luciferase and Renilla luciferase activity was measured by a microplate reader. TALEN-blank vector and pX330 blank vector were used as negative controls. The data represent the mean ± SD of three independent experiments. (*p ≤ 0.05; **p ≤ 0.01; ***p ≤ 0 .001) (**c**) Evaluation of TALEN- and CRISPR/Cas9-mediated DNA cleavage by the T7E1 assay in 293T cells. The endogenous locus was amplified by PCR, and the product was further purified according to the manufacturer’s instructions. The purified PCR product was denatured and reannealed, and the hybridized PCR products were further digested by T7 Endonuclease I. The upper lane shows the separation of the DNA on a 2% agarose gel, this results were cropped from the full-length gels which were presented in Supplementary Figure S5.A and all the gels were run under the same condition; while the lower lane shows relative indel rates in different groups as measured with the ImageJ program. (*p ≤ 0.05; **p ≤ 0.01; ***p ≤ 0 .001) (**d**) Evaluation of TALEN- and CRISPR/Cas9-mediated DNA cleavage by the T7E1 assay in 654hiPS cells. The upper lane shows the separation of DNA on a 2% agarose gel after T7E1 digestion; the lower lane shows the relative indel rates in different groups as measured by ImageJ. (*p ≤ 0.05; **p ≤ 0.01; ***p ≤ 0 .001) (**e**) Sanger sequencing of the different mutant types of HBB intron2 in 293T cells after transfection with either TALEN or CRISPR/Cas9. The blue nucleotides in the upper lane represent two pairs of TALEN recognition sites. The red nucleotides in the lower lane represent the PAM (protospacer adjacent motif) sequence recognized by CRISPR/Cas9.

To induce DSBs near *HBB* loci, we designed two pairs of TALENs and two single--guide RNAs(sgRNA) to directly target the IVS2-654 C > T mutation (Fig. 1a).

We first used a mammalian cell-based single-strand annealing (SSA) assay to determine if the site-specific TALENs and CRISPR/Cas9 disrupted the *HBB* locus in HEK293T cells. The *HBB* intron2 sequence was cloned into the direct repeat half of the luciferase gene. When DSBs were induced by TALENs or CRISPR/Cas9, the stop codon was removed, and an intact luciferase gene was formed via SSA homologous recombination. TALEN-1 induced a 10-fold increase in signal compared to the negative control, while TALEN-2 yielded a 3-fold increase. A 10-fold enhancement of signals was observed for sgRNA-1 while sgRNA-2 produced the highest signals, with an approximately 15-fold change (Fig. 1b). These results demonstrate that the pair of TALENs and two sgRNAs were efficient for *HBB* loci.

To further determine the frequencies of small insertion/deletions (indels) caused by either TALENs or CRISPR/Cas9, we examined *in vivo* DSB efficiency by T7E1(T7 endonuclease I) assay. The TALENs and CRISPR/Cas9 systems were first tested in HEK293T cells. TALEN-1 exhibited much higher DSB efficiency than TALEN-2while sgRNA-2 exhibited much higher efficiency than sgRNA-1 .What’s more, the efficiency of sgRNA-2 was significantly higher than TALEN-1(Fig. 1c). Then, we evaluated the TALENs and CRISPR/Cas9 systems in β-thalassemia patient-derived iPS cells. We observed that TALEN-2 was much more efficient than TALEN-1 while sgRNA-2 had higher efficieny than sgRNA-1. And also, sgRNA-2 yielded higher indel rates than TALEN-1(Fig. 1d). We also cloned the fragment of *HBB* intron2 and subjected it to Sanger sequencing. Our results demonstrated that different indel types formed after TALEN or CRISPR/Cas9 was targeted in 293T cells (Fig. 1e). Because of their higher DSB efficiency, we selected TALEN-1 and sgRNA-2 for the subsequent experiments.

### TALENs mediate higher homologous recombination efficiency than CRISPR/Cas9 in *HBB* loci in iPSCs derived a β-thalassemia patient

**Figure 2:**
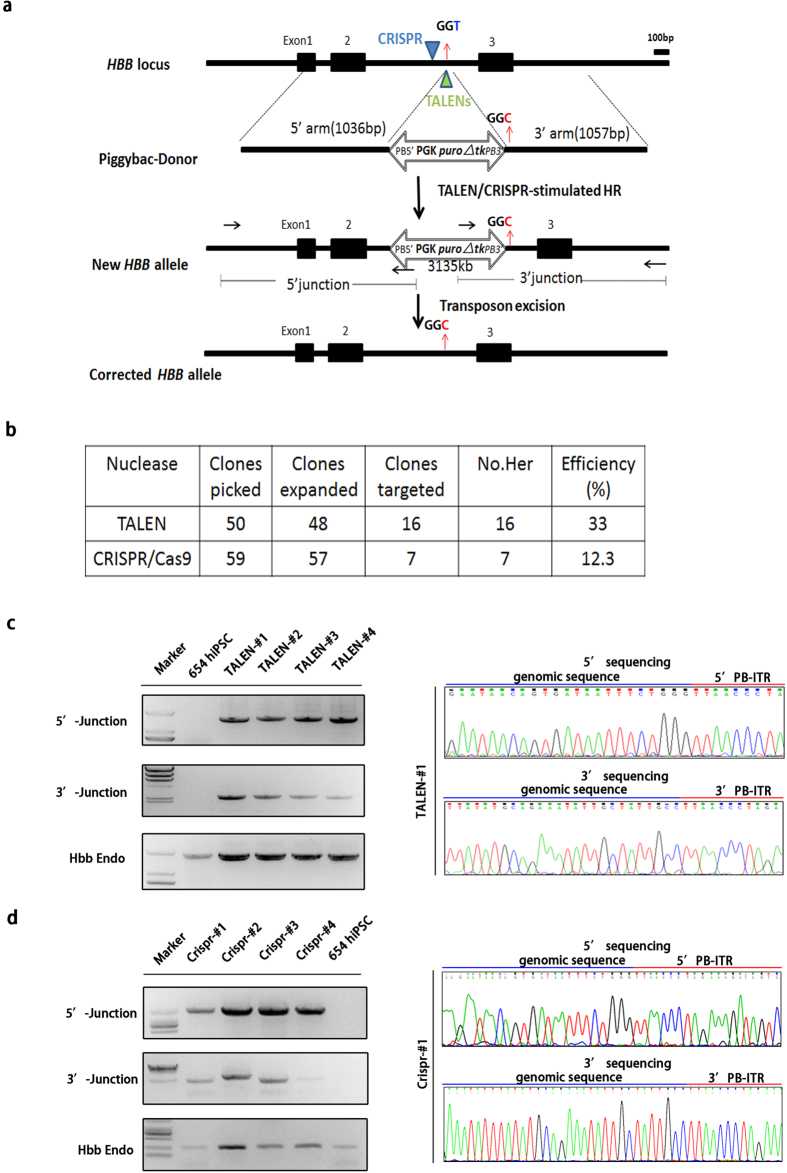
TALENs mediate higher homologous recombination efficiency than CRISPR/Cas9 in *HBB* loci in β-thalassemia patient-derived iPSCs. (**a**) Strategy for gene correction of the IVS2-654 C > T mutation using a TALEN or CRISPR/Cas9 combined with the piggyBac donor vector. A TALEN or CRISPR/Cas9 was used to induce a double-strand break (DSB) near the point mutation. The targeting construct of the piggyBac transposon carrying the selectable marker and flanked by 1000 bp of wild-type genomic sequences was used as a donor vector. In the gene targeting, clones that were correctly integrated into the HBB loci were selected by puromycin and further identified using 5’ junction and 3’ junction PCR primers. (**b**) Summary of successful integration events in TALEN- and CRISPR/Cas9-mediated gene targeting. The expanded clones represent successfully passaged clones, and the targeted clones represent positive clones in both 5’ junction and 3’ junction PCR (Supplementary Figure S2). (**c**) PCR and Sanger sequencing analysis of site-specific homologous recombination mediated by TALENs in β-thalassemia iPSCs. Agorose gel results were cropped from the full-length gels which were presented in Supplementary Figure S5.B and all the gels were run under the same condition. Two pairs of PCR primers (each pair contained one primer corresponding to piggyBac and a second corresponding to HBB outside the targeting construct) were used to detect integration events. Sanger sequencing further confirmed correct integration. (**d**) PCR and Sanger sequencing for analyzing site-specific homologous recombination mediated by CRISPR/Cas9 in β-thalassemia iPSCs. Agorose gel results were cropped from the full-length gels which were presented in Supplementary Figure S5.C and all the gels were run under the same condition.

To correct the IVS2-654 C > T mutation in the *HBB* gene of β-thalassemia derived iPSCs, we constructed a targeting donor vector by inserting two nearly 1-kb segments upstream and downstream of the TTAA sequence at intron2 of *HBB*. The donor vector contains two ITRs of the *piggyBac* transposon and a bi-functional hybrid *puro TK* gene for positive and negative selection^23^. The β-thalassemia iPSCs were first transfected with either TALENs or CRISPR/Cas9 in the presence of donor plasmids. After selection with PUROMYCIN for approximately 12 days, the drug-resistant colonies were isolated, propagated and further tested for homologous recombination by junction PCR amplification using two pairs of primers to screen positive colonies (Fig. 2a).

The targeted clones were positive for amplification with both primer pairs. In the TALENs-mediated targeting group, 16 of 48 clones (33%) exhibited correct homologous recombination, while in the CRISPR/Cas9-mediated targeting group, 7 of 57 clones (12.3%) were positive (Fig. 2b, Supplementary Figure S2). These results indicate that TALENs can mediate a relative high efficiency of homologous recombination in the context of the *HBB* IVS2-654 locus.

We further selected four positive TALENs-targeted clones for detailed analysis. All four cell clones were positive for amplification by the two pairs of junction PCR primers, and Sanger sequencing confirmed accurate homologous recombination (Fig. 2c). We also selected four positive CRISPR/Cas9-targeted clones for further analysis. PCR analysis and subsequent Sanger sequencing confirmed that the IVS2-654 C > T mutation site was corrected in all four CRISPR/Cas9-targeted clones (Fig. 2d). These results suggest that both TALENs and CRISPR/Cas9 can mediate the correction of the IVS2-654 C > T mutation in the *HBB* gene and TALENs seem to mediate higher homologous recombination than CRISPR/Cas9 in this gene context.

### CRISPR/Cas9 displays more obvious potential off-target events than TALENs

To monitor possible off-target events introduced by TALENs cleavage, we analyzed potential gene regions harboring similar recognition and cleavage sites using TAL Effector Nucleotide Targeter 2.0^24^. We then selected ten highly scored sites for further analysis (Table 1). The potential off-target loci of CRISPR/Cas9 were also predicted using the bioinformatics tool Cas-OFFinder^25^. The top ten potential gene loci were selected for analysis in subsequent experiments (Table 2).

**Figure 3:**
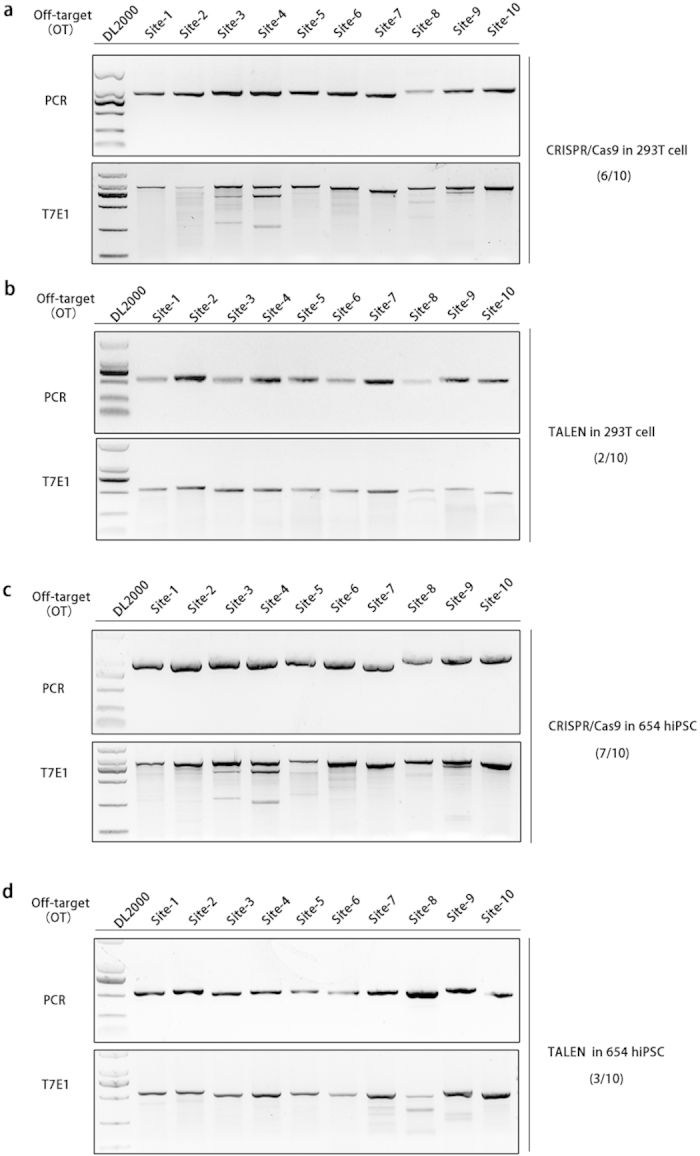
CRISPR/Cas9 displays higher potential off-target efficiency than TALENs. (**a**) T7E1 assay for ten off-target sites potentially recognized by CRISPR/Cas9 in 293T cells. The DNA fragment including the off-target sequence was amplified using appropriate primers. The PCR products were then subjected to the T7E1 assay according to standard protocols. (**b**) T7E1 assay for ten potential off-target sites of TALENs in 293T cells. (**c**) T7E1 assay for ten potential off-target sites of CRISPR/Cas9 in β-thalassemia iPSCs. (**d**) T7E1 assay for ten potential off-target sites of the TALENs in β-thalassemia iPSCs. These agorose gel results were respectively cropped from the full-length gels which were presented in Supplementary Figure S5.D~G and all the gels were run under the same condition.

The potential off-target (OT) events of both TALENs and CRISPR/Cas9 were first measured in 293T cells by the T7E1 assay. We PCR amplified ten potential off-target loci in 293T cells transfected by CRISPR/Cas9. At least six sites (Site-2, 3, 4, 6, 8, 9) had obvious indels (Fig. 3a). PCR amplification and T7E1 were also performed in 293T cells transfected with TALENs, which revealed that only two target sites (Site-8 and 9) had been targeted to some extent (Fig. 3b). These results indicate that CRISPR/Cas9 had more obvious off-target events than TALENs in 293T cells.

To further analyze the safety of TALENs and CRISPR/Cas9 in gene therapy, off-target activity was measured in TALENs- and CRISPR/Cas9-targeted iPS cells mentioned above. In CRISPR/Cas9-targeted iPS cells, we observed clear indels at seven sites (Site-2, 3, 4, 5, 6, 8, 9) (Fig. 3c), while in TALENs-targeted iPS cells, we detected clear indels in three sites (Site-7, 8, 9) (Fig. 3d). These results suggest that CRISPR/Cas9 also had more obvious off target events than TALENs in β-thalassemia iPS cells.

### Gene Corrected β-thalassemia iPSCs retain normal pluripotency

**Figure 4:**
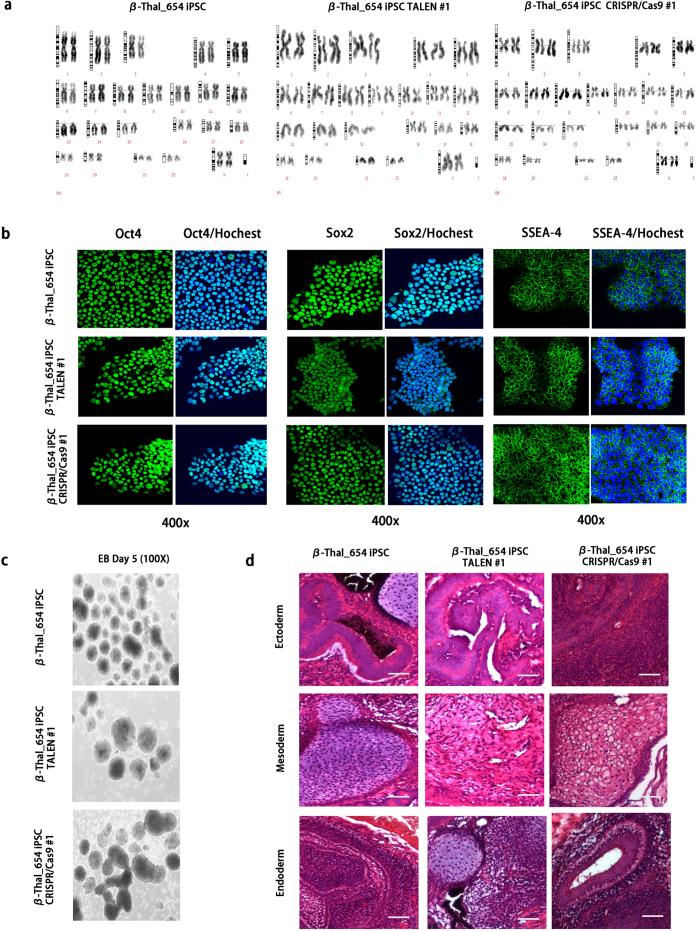
TALEN- or CRISPR/Cas9-targeted iPSCs retain normal pluripotency. (**a**) Karyotyping analysis of the TALEN- and CRISPR/Cas9-targeted β-thalassemia iPSCs. Neither TALEN- nor CRISPR/Cas9-mediated gene editing in β-thalassemia iPSCs caused gross chromosomal alterations. (**b**) Immunostaining of ESC markers (Oct4, Sox2 and SSEA-4) in TALEN- and CRISPR/Cas9-targeted β-thalassemia iPSCs; 400X. Green, antigen staining; Blue, Hoechst. (**c**) EB formation from the TALEN- and CRISPR/Cas9-targeted β-thalassemia iPSCs; 100X Bright field. (**d**) HE staining of teratomas containing tissues of all three germ layers derived from TALEN- and CRISPR/Cas9-targeted β-thalassemia iPSCs; Scale bars, 100 μm.

We selected TALENs- and CRISPR/Cas9-targeted iPSC clones for further characterization. All targeted clones displayed typical iPSC morphology (Supplementary Figure S3) and remained normal karyotypes (Fig. 4a). Immunofluorescence analysis revealed that both targeted clones retained uniform expression of typical pluripotency markers such as OCT4, SOX2, and SSEA-4 (Fig. 4b). Furthermore, TALENs- and CRISPR/Cas9-targeted clones both formed typical embryonic bodies (EB) *in vitro* (Fig. 4c). In addition, to test their pluripotency *in vivo*, the targeted iPS clones were transplanted into severe combined immunodeficiency (SCID) mice, and teratoma formation was observed at 8 weeks. Histological examination revealed that the tumor comprised cell types from all three germ layers in both the TALENs- and CRISPR/Cas9-targeted iPS clones (Fig. 4d). These results suggest that β-thalassemia patient-derived iPSCs retain pluripotency after gene targeting by either TALENs or CRISPR/Cas9.

### Transcription of *HBB* restoration after gene correction

Based on the higher targeting efficiency of TALENs and potential off-target effects of CRISPR/Cas9, we selected TALENs-targeted clone for further hematopoietic differentiation analysis.

**Figure 5:**
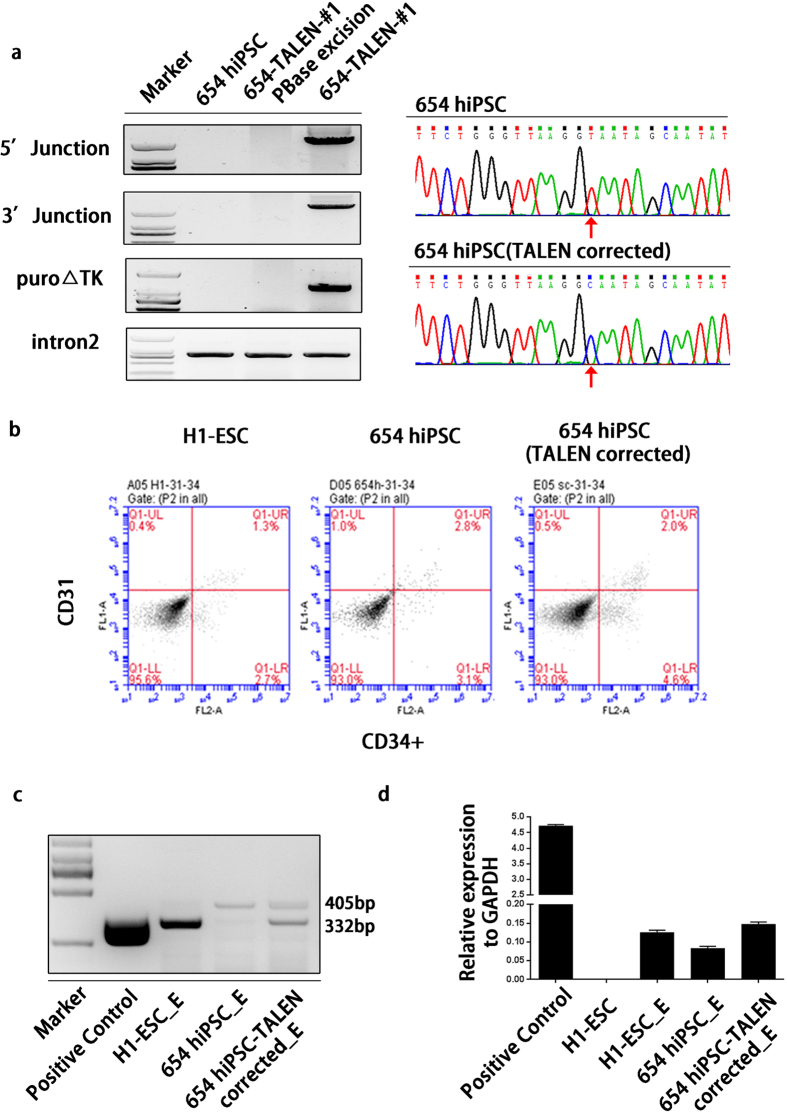
Restoration of HBB transcription after gene correction. (**a**) Agorose gel results show the deletion of selection marker and all the gels were run under the same condition. Primers for the 5’ and 3’ junctions and primers for puro△TK were used to measure the excision of piggyBac. A lack of amplification by both primer pairs indicates no piggyBac reintegration at the targeting site in the β-thalassemia iPS clone. Sanger sequencing confirmed the restoration of normal intron2 after removal. The red arrow indicates the 654C > T mutation. (**b**) Flow cytometric analysis of β-thalassemia hiPS-TALEN-corrected cells, β-thalassemia iPSCs and H1-ESCs using the surface markers CD34 and CD31. (**c**) A conventional RT-PCR assay was performed to amplify HBB cDNA in erythroblasts derived from β-thalassemia hiPS-TALEN corrected cells, β-thalassemia hiPS cells and H1-ESCs. Erythroblasts from Core CD34+ cells were used as a positive control. E: erythroblasts. The agorose gel result was cropped from the full-length gels which was presented in Supplementary Figure S5.H. The normal transcription length is 332 bp, and the transcript length in thalassemia cells was 405 bp. (**d**) RT-PCR analysis of the level of HBB gene expression (relative to GAPDH). Erythroblasts from Cord CD34+ cells were used as a positive control. The values are expressed as the mean ± SD of triplicate samples from a representative experiment.

To remove the drug-selectable cassette from a TALENs-targeted hiPSC clone, the cells were transiently re-expressed the *piggyBac* transposase. After negative selection by 1-(2-deoxy-2-fluoro-1-D-arabinofuranosyl)-5-iodouracil (FIAU), resistant colonies were picked and expanded. Genotyping was performed to detect the deletion of *piggyBac* transposon from the *HBB* locus (Fig. 5a, left panel). Sanger sequencing confirmed the seamless removal of the *piggyBac* transposon based on the restoration of the original intron2 with no exogenous sequence (Fig. 5a, right panel). These iPSC clones were morphologically normal and expressed pluripotency markers correctly(Data not shown).

We employed the OP9 co-culture system to induce the hematopoietic differentiation of β-thalassemia iPSCs before and after gene correction. The morphologies of the TALENs-corrected iPSCs and the parental lines changed rapidly upon differentiation in OP9 co-culture (Supplementary Figure S4). FACS analysis demonstrate that TALENs-corrected iPS cells produced 4.6% hematopoietic progenitor cells (HPCs) detected as CD34+/CD31-, compared to 3.1% in the parental cell lines and 2.7% in H1- embryonic stem (ES) cells (Fig. 5b).

By using conventional RT-PCR to amplify *HBB* cDNA (the forward primer anneals in the first exon of *HBB*, and the reverse primer anneals in the third exon of *HBB*), we confirmed that expression of the wild-type *HBB* cDNA was successfully restored after gene correction (Fig. 5c). We also demonstrated that the expression of the *HBB* gene increased in TALENs-corrected iPSCs-derived erythroblasts compared to uncorrected ones and that the levels were comparable with those in human ESCs-derived erythroblasts (Fig. 5d).

## Discussion

It is the first study to comapre TALENs and CRISPR/Cas9 to directly target the IVS2-654 C > T mutation site in the *HBB* locus, which is widely distributed in China and Southeast Asia. Our findings support the potential of TALENs and CRISPR-Cas9 in β-thalassemia gene correction.

Homologous recombination is a classical method for *in situ* correction of a mutated gene^26^,^27^,^28^. However, this strategy is limited by the relatively low efficiency gene targeting in different cell contexts. Recently, three different systems for specific DNA cleavage to promote efficient homologous recombination have been introduced: ZFNs, TALENs and CRISPR/Cas9^15^,^16^,^17^,^18^. ZFNs are very difficult to design and can yield suboptimal rates of gene targeting^20^. Furthermore, the targeting frequency of ZFNs is approximately 1 target site per 500 bp of DNA, which may hinder ZFN targeting in portions of the genome^29^,^30^. TALENs can be assembled rapidly from freely available modules and have no recognition site limit because TALENs binding sites are located, on average, every 35 bp in the genome^31^. CRISPR/Cas9 relies on a guide RNA to recruit the Cas9 helicase/nuclease to the target site, with target sites at an average interval of 8 bp in the genome^17^,^18^. Most gene correction requires the disruption to be near the mutation site, and thus TALENs and CRISPR/Cas9 should be better choice to perform DNA cleavage near the gene mutation site. From different angles, TALENs and CRISPR-Cas9 show different characteristics. Recently,it is reported that CRISPR-Cas9 exhibited higher making double strand break efficiency than TALENs while TALENs had higher homologous recombination in AAVS1 and AAT2 loci^32^. And there is also report that CRISPR-Cas9 show abvious off target events in HBB loci^33^.

Both TALENs and CRISPR/Cas9 combined with donor DNA provide a good strategy to cure the gene mutation *in situ* in β-thalassemia-derived iPSCs. TALENs and CRISPR/Cas9 have each been reported to mediate gene correction in β-thalassemia-derived iPSCs^2^,^21^,^22^. However, there has been no systematic comparison of TALENs and CRISPR/Cas9 in the direct targeting of point mutations in the *HBB* gene, such as the IVS2-654 C > T mutation, in β-thalassemia-derived iPSCs. In this study, we designed efficient TALENs and CRISPR/Cas9 systems to directly target the mutation site IVS2-654. The efficient CRISPR/Cas9 exhibited higher DSB efficiency than TALENs, consistent with previous reports for other gene loci^32^,^34^. The quantitative results demonstrate that the efficiency of TALENs in facilitating targeted integration events was higher than that of CRISPR/Cas9 in the specific IVS2-654 loci (TALENs: 33% vs. CRISPR/Cas9: 12.3%). Single-strand DNA breaks reportedly stimulate efficient homologous recombination without inducing the error-prone non-homolous-end-joining pathway^35^. TALENs typically generate DSBs with single-strand overhangs in the space between the two TALE-binding sites, while CRISPR/Cas9 reportedly produces blunt-end DSBs^36^,^37^. Recently,Cas9 nickase was also reported to mediate higher homologous recombination efficiency due to the producing single-strand overhangs^35^,^38^,^39^. The different types of DSBs generated by TALENs and CRISPR/Cas9 may result in different preferences for the repair pathway. Although TALENs and CRISPR have been reported to have comparable abilities to facilitate integration efficiency^32^ , our results suggest that TALENs exhibit higher gene integration efficiency than CRISPR/Cas9 at the specific *HBB* IVS2-654 gene locus in β-thalassemia-derived iPSCs.

The risk of off-target mutagenesis is one of the most important obstacles to the therapeutic use of programmable nucleases. According to a recent report^40^, both TALENs and CRISPR/Cas9 can bind to DNA despite a few base mismatches. In this study, we predicted the most likely potential off-target sites of TALENs and CRISPR/Cas9 using widely accepted bioinformatics tools. The T7E1 assay revealed that CRISPR/Cas9 induced obvious indel formation at six other sites, while TALENs induced slight indel formation at two other sites in 293T cells. Although a high risk of off-target events of CRISPR/Cas9 has been reported in certain cell types^33^,^41^,^42^, whole genome-wide sequencing studies have demonstrated that both TALENs and CRISPR/Cas9 have minimal off-target effects in iPSCs after gene targeting^43^,^44^. Cas9 nickase had reported to display much lower off-target effect than the wild type Cas9 and it may provide an alternative choice for precise gene correcton^45^,^46^,^47^. Although completely ruling out other genomic changes requires whole-genome sequencing, the current study suggests that TALENs may be a good choice when targeting the *HBB* IVS2-654 gene locus in β-thalassemia iPSCs due to the relatively higher off-target effects of CRISPR/Cas9 compared to TALENs.

The combination of nucleases (TALENs or CRISPR/Cas9) and the piggyBac system provides an ideal gene correction strategy by achieving site-specific correction without any footprint, and this strategy has been widely used in various gene therapy case studies^10^,^22^,^48^,^49^. In this study, we obtained the TALENs-corrected iPSC clone with seamless removal of the *piggyBac* transposon, and we detected an increase in the transcription of the *HBB* gene by qRT-PCR in erythroid cells differentiated from TALENs-corrected iPSCs compared to those obtained from parental iPSCs. However, transplantability and achieving a complete globin-switch in iPSC hematopoietic differentiation remain to be achieved. Therefore, further studies need to focus on the generation of functional, mature, transplantable hematopoietic progenitor cells from TALENs- or CRISPR/Cas9-corrected β-thalassemia iPSCs.

## Materials and Methods

### Animals

All mouse experimental procedures were approved by the the Institutional Animal Care and Use Committee at Peking Union Medical College & Chinese Academy of Medical Sciences (No. ACUC-A01-2013-029). And all animal care and experiments were carried out in accordance with the institutional ethical guidelines for animal experiments.

### TALENs, CRISPR/Cas9 and piggyBac donor vector construction

The TALENs were designed and constructed by ViewSolid (Beijing, China). The full amino acid sequences of the TALENs are provided in the supporting material (Supplementary Figure S1). CRISPR/Cas9 vector pX330 was purchased from Addgene (Ad42230). Guide RNAs were designed according to the rule of 5’-GN20NGG-3’. The guide RNA oligonucleotides were purchased from Invitrogen and inserted into pX330 according to the manufacturer’s protocol. To construct the donor plasmid, we first amplified 1000-bp genomic sequence fragments near the IVS2-654 site in intron 2. We deleted the OSKM cassette from *piggyBac*-OSKM (a gift from Dr.Allan Bradley) and named the resulting construct *piggyBac*-puro△TK. Then, we inserted two homology arms into *piggyBac*-puro△TK using one-step cloning methods.

### Cell culture

293T cells were maintained in DMEM supplemented with 10% fetal bovine serum (FBS). The β-thalassemia patient iPSCs were kindly provided by the lab of Pan Guan Jing^2^. Human iPSCs were cultured in mTeSR1 medium on Matrigel-coated 6-well plates. The medium was changed daily.

### Single-strand annealing assay

The SSA luciferase reporter pSSA-HBB-IVS2 plasmid was constructed by ViewSolid. Briefly, a TALEN and CRISPR/Cas9 target sequence and a stop codon were inserted into the direct repeat halves of the firefly luciferase gene, and the vector was named pSSA-HBB-IVS2. Next, 400 ng of TALEN plasmid or 400 ng of Cas9 and gRNA plasmid, 100 ng of pSSA-HBB-IVS2, and 25 ng of pRL-TK-*Renilla* luciferase (Promega) were co-transfected into HEK293 cells in 24-well plates. At 48 h after transfection, the firefly luciferase and *Renilla* activities were determined according to the protocol of the Dual-Luciferase Reporter Assay System (Promega). Luciferase activity was monitored with a microplate luminometer (Promega). All experiments were repeated three times.

### T7E1 assay for on/off-target analysis

Genomic DNA was isolated from cells transfected with TALENs, CRISPR/Cas9 or control plasmids using a DNA extraction kit (Promega) according to the manufacturer’s instructions. PCR to amplify endogenous loci was performed for 35 cycles, as described above, and the fragments were purified using the Omega gel kit according to the manufacturer’s instructions. One microgram of purified PCR product was denatured and reannealed in NEBuffer 2 (New England Biolabs) using a thermocycler with the following protocol: 95 °C, 5 min; 95–85 °C at −2 °C/s; 85–25 °C at −0.1 °C/s; hold at 4 °C. Hybridized PCR products were treated with 10 U of T7 Endonuclease I at 37 °C for 60 min in a reaction volume of 20 μl. ImageJ was used to measure indel formation by measuring the intensities of the bands separated by electrophoresis on a 2% agarose gel. The following formula was used to calculate the percentage of indel formation: % indel formation = 100 × [1 - (1 - fraction cleaved)1/2]; fraction cleaved = 100 × sum of the cleavage product peak/(cleavage product + parent peak).

### Gene targeting by TALEN and CRISPR/Cas9 in iPSCs

For gene targeting, 2 × 10^6^ iPSCs were electroporated with CRISPR/Cas9 or TALEN pairs with Donor vector. Then, the cells were plated onto Matrigel-coated 6-well plates in the presence of Y-27632 (10 μM, Sigma) for 1 day. Positive clones were selected by puromycin (0.5 μg/ml) in mTeSR1. The selected colonies were verified by genomic PCR. PCR was performed using LA Taq (Takara) according to the manufacturer’s instructions. In all reactions, 100 ng of genomic DNA was used as the template. A primer set including 5Junction-F (on the *HBB* locus, upstream of the 5’ homology arm) and 5Junction-R (in the drug resistance cassette) was used to amplify a 2.4-kb product of the 5’ junction of a targeted integration. A primer set including 3Junction-F (in the drug resistance cassette) and 3Junction-R (downstream of the 3’ homology arm) was used to amplify a 2.7-kb product.

### Karyotype analysis

Metaphase spreads were prepared from cells treated with 50 ng/mL colcemid for 6 h, followed by the standard protocol for high-resolution G binding. Twenty chromosome spreads were examined for each sample.

### Immunofluorescence staining

Immunofluorescence (IF) staining was performed using primary antibodies (all at 1:200 dilutions) to detect Oct4 (Abcam), Sox2 (Abcam) and SSEA-4 (Santa Cruz Biotechnology). Nuclei were counterstained with Hoechst stain (Sigma).

### Teratoma formation

Cells from a confluent 10-cm plate were harvested by digestion with 2 mg/ml dispase, resuspended in Matrigel and injected subcutaneously into immunodeficient mice. Eight weeks after injection, teratomas were dissected, fixed in 4% paraformaldehyde, and processed for hematoxylin/eosin (HE) staining.

### Excision of the *piggyBac* transposon cassette

To remove the *piggyBac* cassette, 3 × 10^6^ corrected iPSCs were transfected with 10 mg of hyperactive transposase vector, hyperPBase (a gift from Dr. Allan Bradley), followed by selection with FIAU (0.5 mM) for 7 d. To verify their excision, primers for amplifying puroΔTK and primers for integration events were used to detect the removal of the *piggyBac* transposon cassette from the genome. These primers are also listed in the supplemental material.

### Hematopoietic differentiation of human iPSCs

Human iPS cells were harvested by treatment with 2 mg/ml dispase (Invitrogen) and co-cultured with OP9 stromal cells at an approximate density of 5 × 10^6^/20 ml per 10 cm dish in 20 ml of α-MEM (GIBCO) supplemented with 10% fetal bovine serum (FBS; HyClone, Logan, Utah), 100 mM monothioglycerol (MTG; Sigma, St. Louis, MO), and 100 μM vitamin C. The co-cultures of OP9 with pluripotent cells were incubated for 8 days, with replacement of half of the medium on days 4 and 6. Differentiated hiPSCs were harvested at day 8. CD34+ cells were sorted out using the direct CD34 Progenitor Cell Isolation Kit (Miltenyi Biotech, Auburn, CA).

## Additional Information

**How to cite this article**: Xu, P. *et al.* Both TALENs and CRISPR/Cas9 directly target the *HBB* IVS2-654 (C >T) mutation in β-thalassemia-derived iPSCs. *Sci. Rep.* **5**, 12065; doi: 10.1038/srep12065 (2015).

## Supplementary Material

Supplementary Information

## Figures and Tables

**Table 2:** The top 10 potential off-target sites of CRISPR/Cas9 for the recognition of HBB IVS-2 654 loci.

Serial number	Gene name	Sequence	Orientation	No. of mismatches
on target	HBB	CAGTGATAATTTCTGGGTTAAGG	−	0
Site-1	HHAT	CAGTGATtATTTCTGGGTTATGG	−	1
Site-2	NTRK2	CAGTGATAATTTCaGGGgTATGG	+	2
Site-3	ATXN10	gAGTGATgATTTCTGGGTTAAGG	+	2
Site-4	TPRG1	CAGTGATtATTTCTGGGTggTGG	+	3
Site-5	EHF	tAGTGATAgTTTCTGGGTgAAGG	+	3
Site-6	CCDC178	CtaTGATAATTTCTtGGTTAGGG	+	3
Site-7	CDC7	CAGTGtTAATTTCTGttTTATGG	−	3
Site-8	RNF216	CAGTGActATTTCTGGGTaAGGG	+	3
Site-9	CNTNAP2	gAaTGATAcTTTCTGGGTTAGGG	−	3
Site-10	C2orf73	CAGTGgTAATTTCaGaGTTAAGG	−	3

Lowercase nucleotides represent a mismatch compared with the on-target sequences.

**Table 1:** The top 10 potential off-target sites of TALENs for the recognition of HBB IVS-2 654 loci.

Serial number	Gene name	TAL 1 Target	TAL 2 Target	Spacer Length
on target	HBB	T ATATGCAGAGATATT	T AACAGTGATAATTTCT	16
Site-1	C4orf21	T AAAAAAAGAAAAATT	T ATATACAAAAATATT	20
Site-2	TTLL7	T AGATGCAAAAATCCT	T ATATCCACAAATATT	25
Site-3	TEX41	T GAAAATCATAATCTCT	T AAATACAGAAATATT	18
Site-4	EGFR	T ATATGTACAAATAAA	T ATATACAAAAATATA	22
Site-5	CTNNA3	T ATATGCCGAAATATT	T AAAACTAATCACTTCT	25
Site-6	SMEK2	T ATATACATAAATATT	T TACAATGCTAATTTAT	25
Site-7	PTPRD	T ATATACAAACATATT	T ATATGCACATATATG	20
Site-8	GPR98	T AACAATAATAATCTCT	T ATATACACACTCATT	26
Site-9	SLC9A6	T ATATGTAGATATATA	T ATATGCAGAAATATG	30
Site-10	TTC28	T ATATGCAGAAATATG	T AATAGATATAACTTCT	25
