# Supplementary material for: Both TALENs and CRISPR/Cas9 directly target the HBB IVS2–654 (C > T) mutation in β-thalassemia-derived iPSCs
Source: Sci Rep. 2015 Jul 9;5:12065. doi: 10.1038/srep12065 (PMC4496796; doi:10.1038/srep12065)
Supplement: Supplementary Information [file srep12065-s1.pdf]

# Both TALENs and CRISPR/Cas9 directly target the *HBB* IVS2–654 (C>T) mutation in β-thalassemia-derived iPSCs

Peng Xu[1,2#], Ying Tong[1,2#], Xiu-zhen Liu[1,2], Ting-ting Wang[1,2], Li Cheng[1,3], Bo-yu Wang[1,2], Xiang Lv[1,2], Yue Huang[1,3], De-pei Liu[1,2]*

[1]*State Key Laboratory of Medical Molecular Biology, Institute of Basic Medical Sciences, Chinese Academy of Medical Sciences and Peking Union Medical College, Beijing, China*

[2] *Department of Biochemistry and Molecular Biology, Institute of Basic Medical Sciences, Chinese Academy of Medical Sciences and Peking Union Medical College, Beijing, China*

[3]*Department of Medical Genetics, Institute of Basic Medical Sciences, Chinese Academy of Medical Sciences, School of Basic Medicine Peking Union Medical College, Beijing, China*

# These authors contributed equally to this study

***Correspondence**: De-pei Liu, PhD, State Key Laboratory of Medical Molecular Biology, Institute of Basic Medical Sciences, Chinese Academy of Medical Sciences and Peking Union Medical College, 5 Dong Dan San Tiao, Beijing 100005, P.R. China. Telephone: 86-10-69156415; Fax: 86-10-69153086; Email: liudp@pumc.edu.cn

Supplementary information are as follows:

Figure S1. TALENs, CRISPR/Cas9 and piggyBac donor vector construction.

Figure S2. PCR identification of TALEN- and CRISPR/Cas9-mediated gene targeting in 654-hiPSC.

Figure S3. Human stem cell morphology of β-thalassemia patient-derived iPSCs.

Figure S4. Morphology change upon hematopoietic differentiation in OP9 co-culture.

Figure S5. Full-length gels which have been cropped in the main text are presented.

Table S1. Primer sequence summary.

Figure S1

A.

TALEN-1- L-protein sequence: 960aa

MAPKKKRKVDYKDHDGDYKDHDIDYKDDDDKGTVDLRTLGYSQQQQEKIKPKVRSTVAQHHEALV
GHGFTHAHIVALSQHPAALGTVAVKYQDMIAALPEATHEAIVGVGKQWSGARALEALLTVAGELRGPPLQ
LDTGQLLKIAKRGGVTAVEAVHAWRNALTGAPLNLTPEQVVAIASNIGGKQALETVQRLLPVLCQAHGLTP
DQVVAIASNIGGKQALETVQRLLPVLCQAHGLTPDQVVAIASHDGGKQALETVQRLLPVLCQAHGLTPAQ
VVAIASNIGGKQALETVQRLLPVLCQAHGLTPDQVVAIASNNGGKQALETVQRLLPVLCQAHGLTPDQVV
AIASNGGGKQALETVQRLLPVLCQAHGLTPAQVVAIASNNGGKQALETVQRLLPVLCQAHGLTPDQVVAI
ASNIGGKQALETVQRLLPVLCQAHGLTPDQVVAIASNGGGKQALETVQRLLPVLCQAHGLTPAQVVAIAS
NIGGKQALETVQRLLPVLCQAHGLTPDQVVAIASNIGGKQALETVQRLLPVLCQAHGLTPDQVVAIASNG
GGKQALETVQRLLPVLCQAHGLTPAQVVAIASNGGGKQALETVQRLLPVLCQAHGLTPAQVVAIASNGG
GKQALETVQRLLPVLCQAHGLTPAQVVAIASHDGGKQALETVQRLLPVLCQAHGLTPAQVVAIASNGGGR
PALESIVAQLSRPDPALAALTNDHLVALACLGGRPALDAVKKGLPHAPALIKRTNRRIPERTSHRVAGSQLVK
SELEEKKSELRHKLKYVPHEYIELIEIARNPTQDRILEMKVMEFFMKVYGYRGEHLGGSRKPDGAIYTVGSPI
DYGVIVDTKAYSGGYNLPIGQADAMQSYVEENQTRNKHINPNEWWKVYPSSVTEFKFLFVSGHFKGNYK
AQLTRLNHITNCNGAVLSVEELLIGGEMIKAGTLTLEEVRRKFNNGEINF*

TALEN-1- R-protein sequence: 960aa

MAPKKKRKVDYKDHDGDYKDHDIDYKDDDDKGTVDLRTLGYSQQQQEKIKPKVRSTVAQHHEALV
GHGFTHAHIVALSQHPAALGTVAVKYQDMIAALPEATHEAIVGVGKQWSGARALEALLTVAGELRGPPLQ
LDTGQLLKIAKRGGVTAVEAVHAWRNALTGAPLNLTPEQVVAIASNIGGKQALETVQRLLPVLCQAHGLTP
DQVVAIASNGGGKQALETVQRLLPVLCQAHGLTPAQVVAIASNIGGKQALETVQRLLPVLCQAHGLTPDQ
VVAIASNGGGKQALETVQRLLPVLCQAHGLTPAQVVAIASNNGGKQALETVQRLLPVLCQAHGLTPDQV
VAIASHDGGKQALETVQRLLPVLCQAHGLTPAQVVAIASNIGGKQALETVQRLLPVLCQAHGLTPDQVVAI
ASNNGGKQALETVQRLLPVLCQAHGLTPDQVVAIASNIGGKQALETVQRLLPVLCQAHGLTPDQVVAIAS
NIGGKQALETVQRLLPVLCQAHGLTPDQVVAIASNIGGKQALETVQRLLPVLCQAHGLTPDQVVAIASNG
GGKQALETVQRLLPVLCQAHGLTPAQVVAIASNIGGKQALETVQRLLPVLCQAHGLTPDQVVAIASNGGG
KQALETVQRLLPVLCQAHGLTPAQVVAIASNGGGKQALETVQRLLPVLCQAHGLTPAQVVAIASNNGGKQ
ALETVQRLLPVLCQAHGLTPDQVVAIASHDGGRPALESIVAQLSRPDPALAALTNDHLVALACLGGRPALDA
VKKGLPHAPALIKRTNRRIPERTSHRVAGSQLVKSELEEKKSELRHKLKYVPHEYIELIEIARNPTQDRILEMKV
MEFFMKVYGYRGEHLGGSRKPDGAIYTVGSPIDYGVIVDTKAYSGGYNLPIGQAREMQRYVEENQTRNK
HINPNEWWKVYPSSVTEFKFLFVSGHFKGNYKAQLTRLNHITNCNGAVLSVEELLIGGEMIKAGTLTLEEVR
RKFNNGEINF*

B.

spCas9 protein sequence:1503aa

MDYKDHDGDYKDHDIDYKDDDDKMAPKKKRKVGIHGVPAADKKYSIGLDIGTNSVGWAVITDEYKVPSKKFK
VLGNTDRHSIKKNLIGALLFDSGETAEATRLKRTARRRYTRRKNRICYLQEIFSNEMAKVDDSFFHRLEESFLVEED
KKHERHPIFGNIVDEVAYHEKYPTIYHLRKKLVDSTDKADLRLIYLALAHMIKFRGHFLIEGDLNPDNSDVDKLFIQ
LVQTYNQLFEENPINASGVDAKAILSARLSKSRRLENLIAQLPGEKKNGLFGNLIALSLGLTPNFKSNFDLAEDAKL
QLSKDTYDDDLDNLLAQIGDQYADLFLAAKNLSDAILLSDILRVNTEITKAPLSASMIKRYDEHHQDLTLLKALVR

QQLPEKYKEIFFDQSKNGYAGYIDGGASQEEFYKFIKPILEKMDGTEELLVKLNREDLLRKQRTFDNGSIPHQIHL
GELHAILRRQEDFYPFLKDNREKIEKILTFRIPYYVGPLARGNSRFAWMTRKSEETITPWNFEEVVDKGASAQSFI
ERMTNFDKNLPNEKVLPKHSLLYEYFTVYNELTKVKYVTEGMRKPAFLSGEQKKAIVDLLFKTNRKVTVKQLKED
YFKKIECFDSVEISGVEDRFNASLGTYHDLLKIIKDKDFLDNEENEDILEDIVLTLTLFEDREMIEERLKTYAHLFDDK
VMKQLKRRRYTGWGRLSRKLINGIRDKQSGKTILDFLKSDGFANRNFMQLIHDDSLTFKEDIQKAQVSGQGDSL
HEHIANLAGSPAIKKGILQTVKVVDELVKVMGRHKPENIVIEMARENQTTQKGQKNSRERMKRIEEGIKELGSQI
LKEHPVENTQLQNEKLYLYYLQNGRDMYVDQELDINRLSDYDVDHIVPQSFLKDDSIDNKVLTRSDKNRGKSDN
VPSEEVVKKMKNYWRQLLNAKLITQRKFDNLTKAERGGLSELDKAGFIKRQLVETRQITKHVAQILDSRMNTKY
DENDKLIREVKVITLKSKLVSDFRKDFQFYKVREINNYHHAHDAYLNAVVGTALIKKYPKLESEFVYGDYKVYDVR
KMIAKSEQEIGKATAKYFFYSNIMNFFKTEITLANGEIRKRPLIETNGETGEIVWDKGRDFATVRKVLSMPQVNIV
KKTEVQTGGFSKESILPKRNSDKLIARKKDWDPKKYGGFDSPTVAYSVLVVAKVEKGKSKKLKSVKELLGITIMERS
SFEKNPIDFLEAKGYKEVKKDLIIKLPKYSLFELENGRKRMLASAGELQKGNELALPSKYVNFLYLASHYEKLKGSP
EDNEQKQLFVEQHKHYLDEIIEQISEFSKRVILADANLDKVLSAYNKHRDKPIREQAENIIHLFTLTNLGAPAAFKY
FDTTIDRKRYTSTKEVLDATLIHQSITGLYETRIDLSQLGGDKRPAATKKAGQAKKKK.EFLELADQPRLCLLVASHL
LFAPPPCLP.PWKVPLPLSFPNKMRKLHRIV.VGVILFWGVGWGRTARGRIGKRIAGMLG

C.

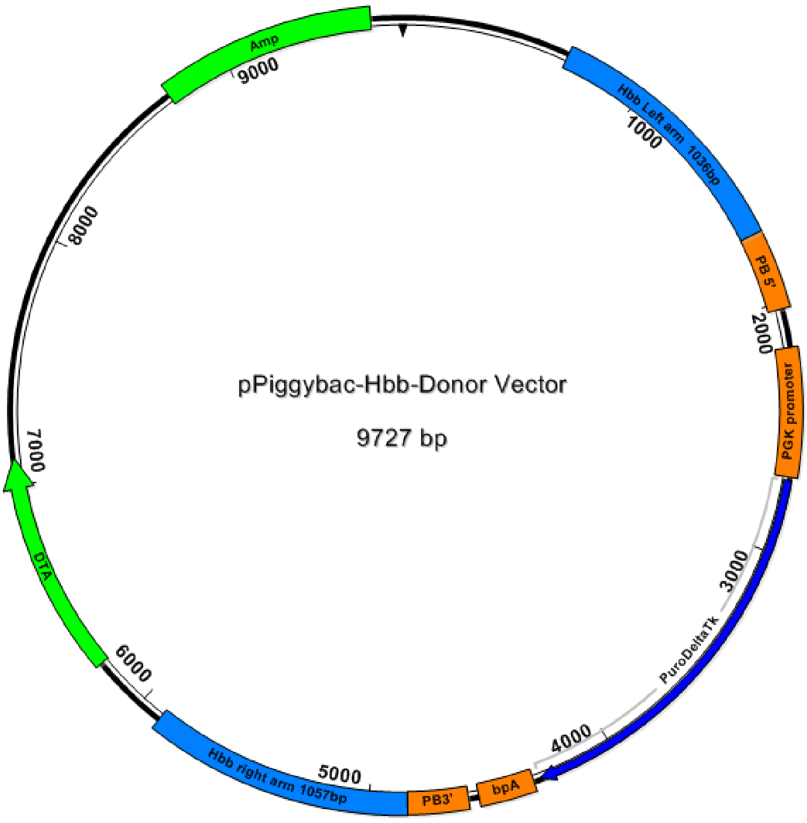

Figure S1 **TALENs, CRISPR/Cas9 and *piggyBac* donor vector construction**

A. The amino acid sequences of TALEN-1 for targeting HBB IVS-2 654 loci
B. The amino acid sequences of Cas9 from px330 for targeting HBB IVS-2 654 loci
C. The vector map of piggyBac donor vector.

Figure S2

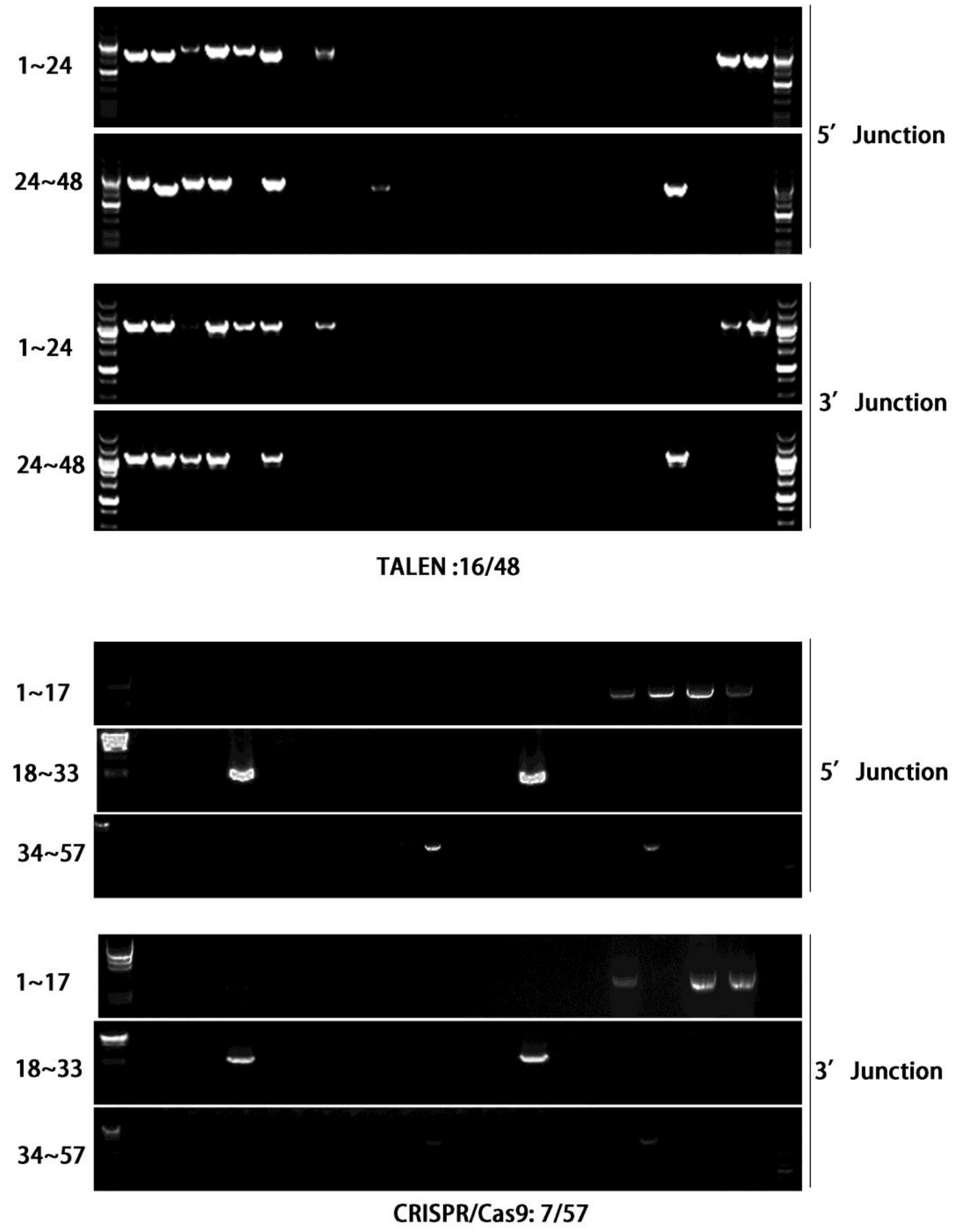

Figure S2 **PCR identification of TALENs and CRISPR/Cas9 mediated gene targeting in 654hiPSC.** After gene targeting, clones with correctly integrating into the HBB loci was selected by puromycin and further identified by 5' junction and 3' junction PCR primers. The upper lane showed the PCR gel results in TALEN targeted 48 cell clones; while the down lane showed the PCR results in CRISPR/Cas9 targeted 57 cell clones.

Figure S3

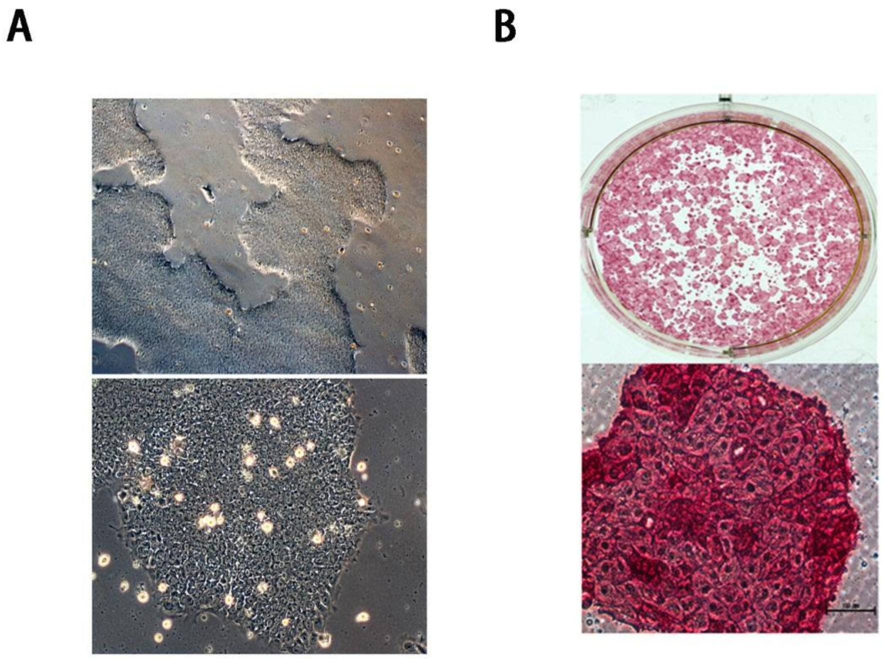

Figure S3 **Human pluriptent stem cell morphology of β-thalassemia patient derived iPSC**

A. The morphology in bright field by respectively 50x (up) and 200x (down) in β -thalassemia iPS cells
B. AP (alkaline phosphatase) staining showed the typical human stem cell morphology by respectively 50x (up) and 200x(down) in β-thalassemia iPS cells

Figure S4

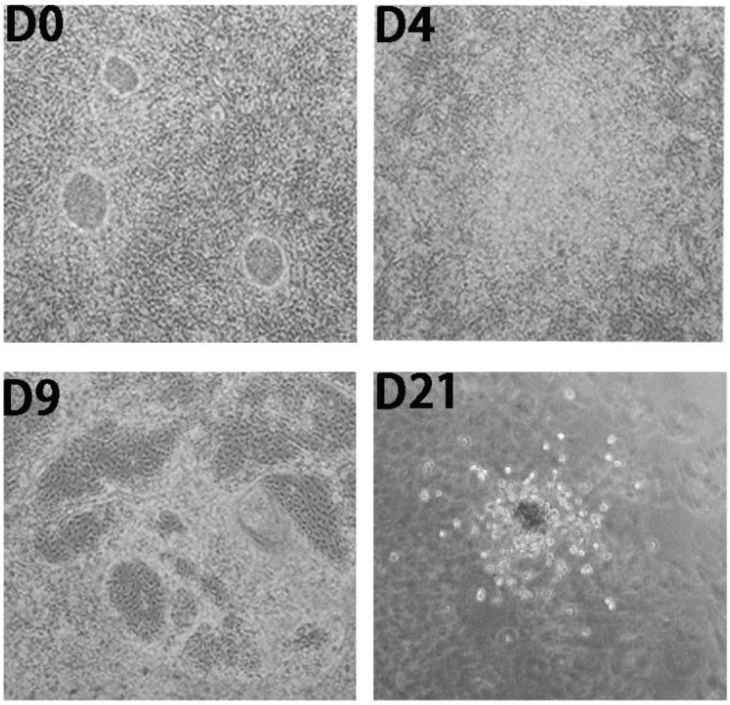

Figure S4 **Morphology change rapidly upon hematopoietic differentiation by using OP9 co-culture.** Human iPSCs were digested and cocultured with overgrown OP9; After 9 d of coculture, differentiated blood-forming hPSC colonies with radial sac-like structures are formed; upon hematopoietic differentiation at D21, the cell showed erythrobalst progenetor morphology (D represents day).

Figure S5

A

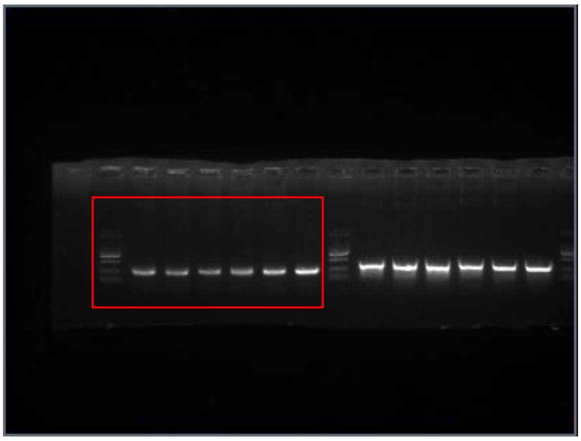

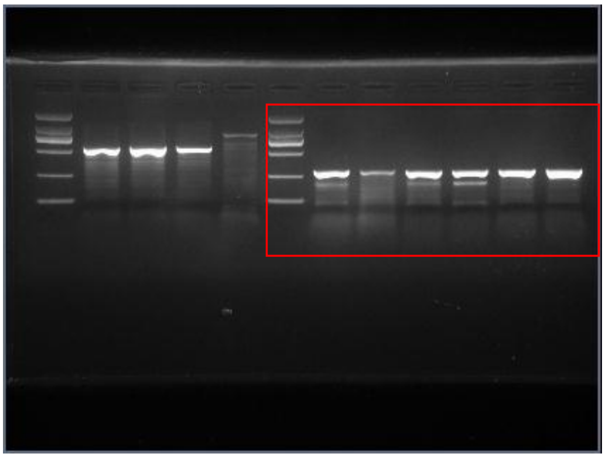

B

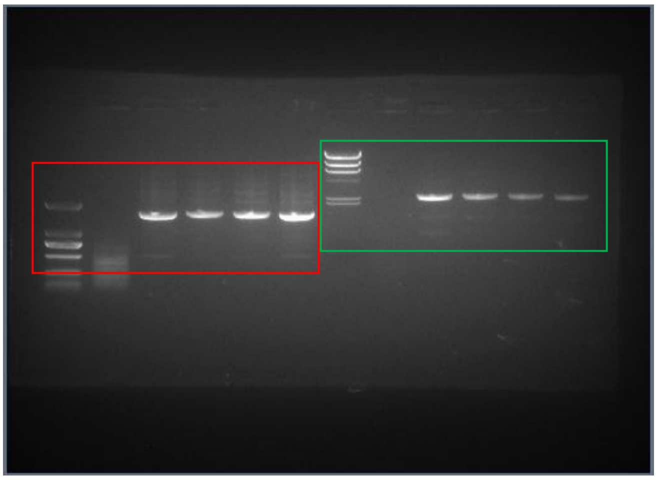

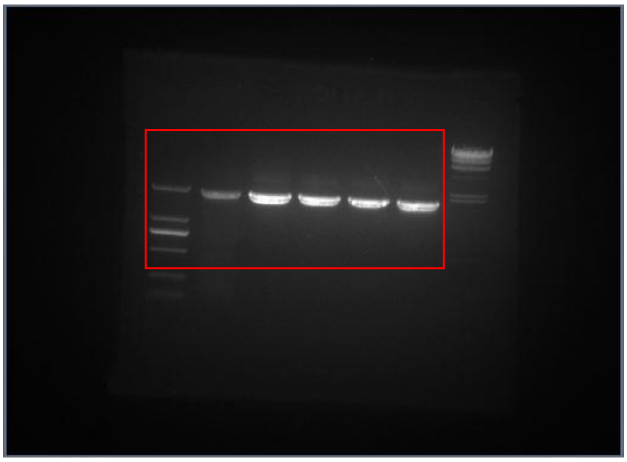

C

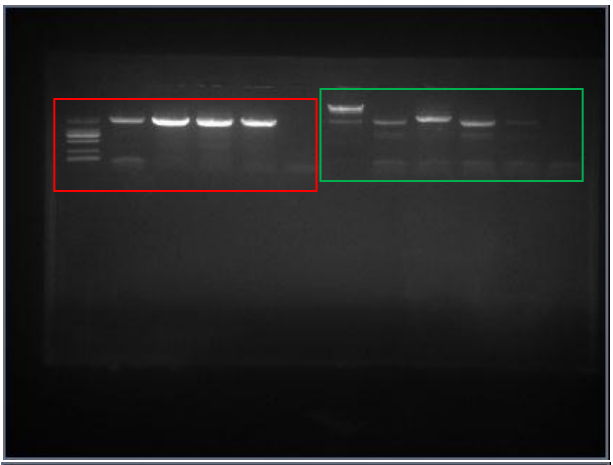

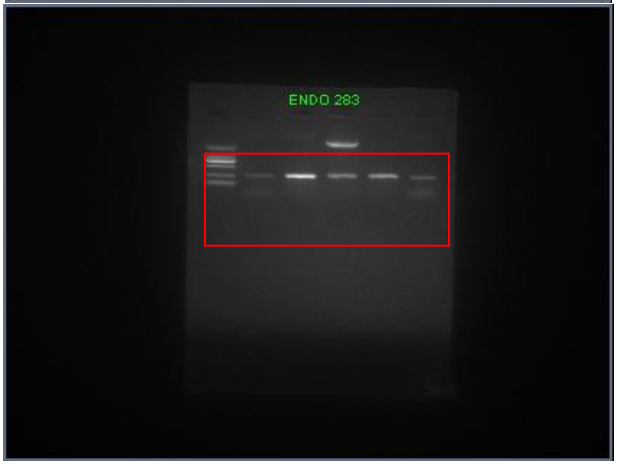

D

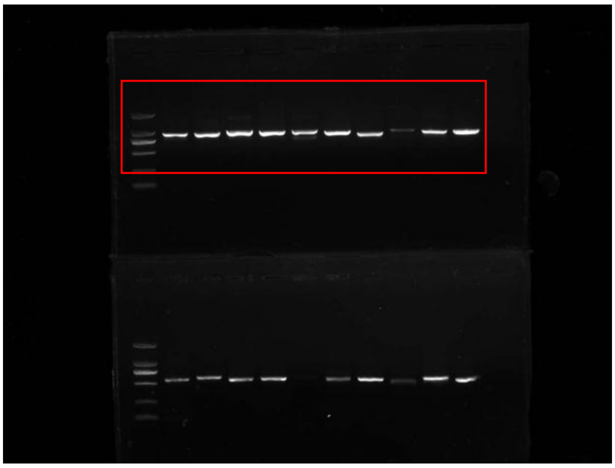

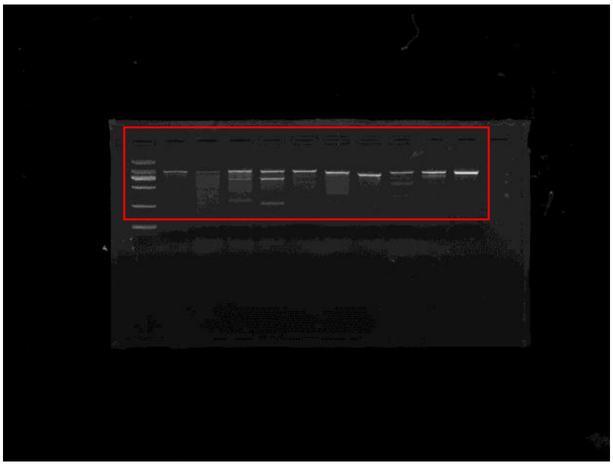

E

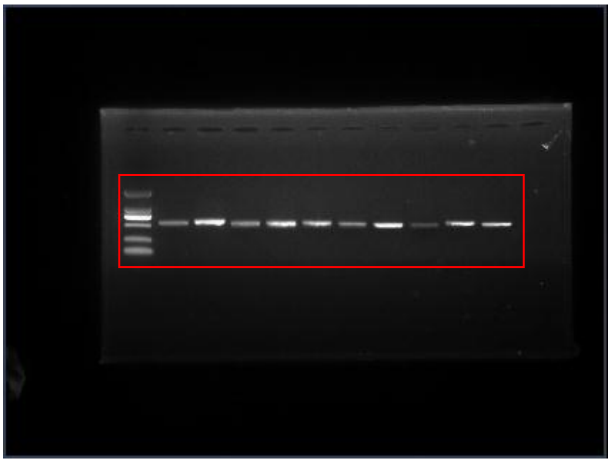

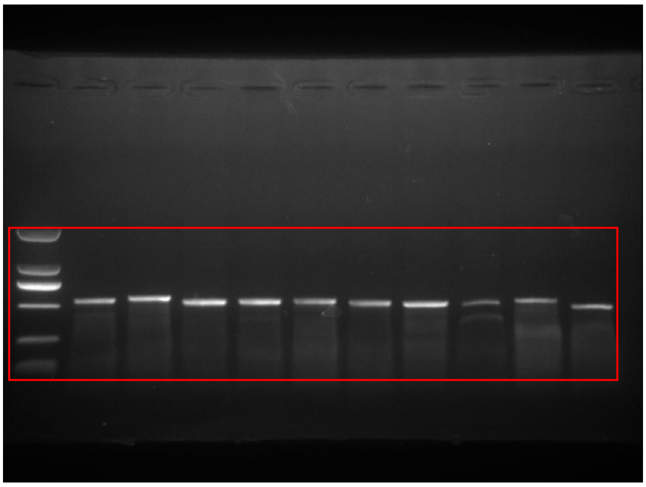

F

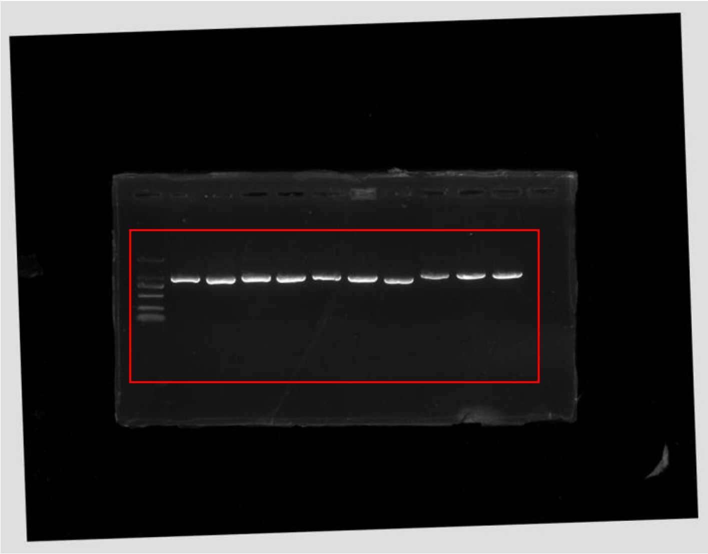

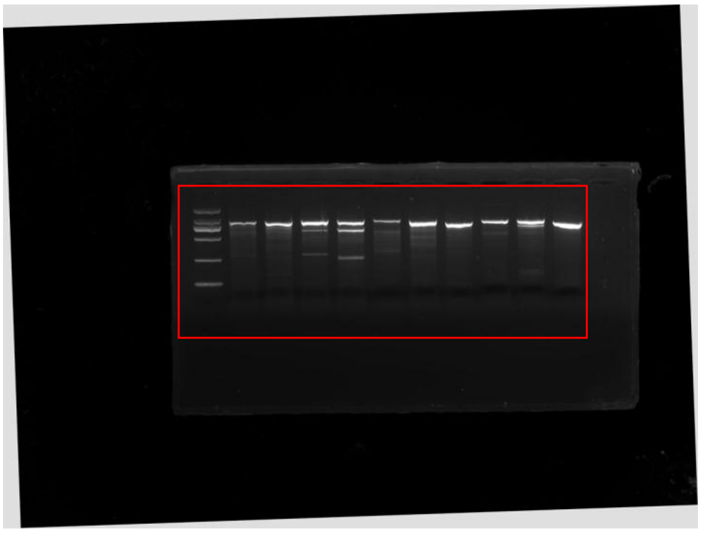

G

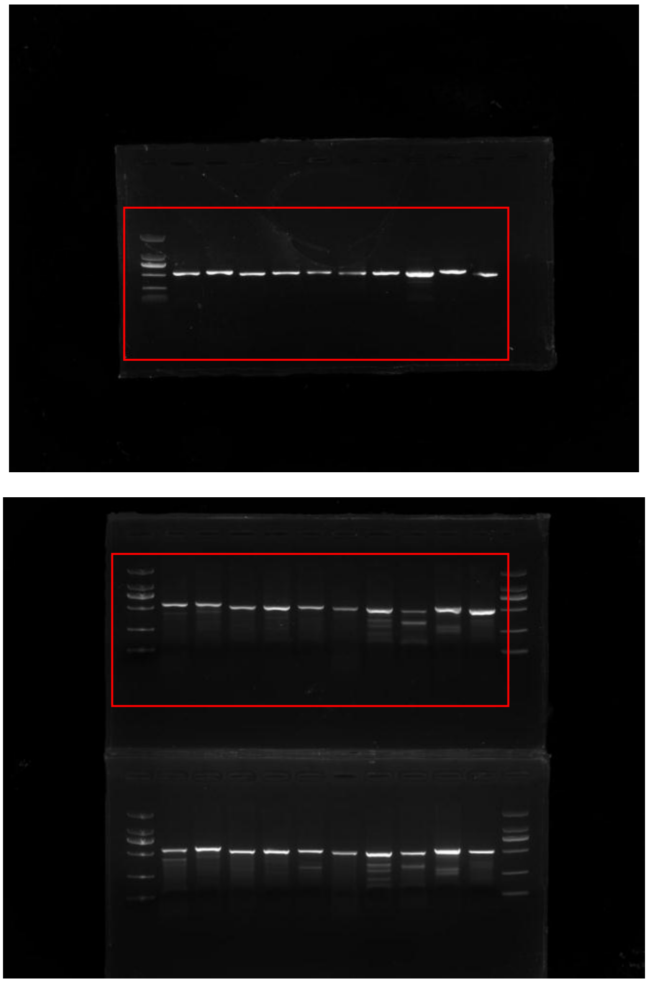

H

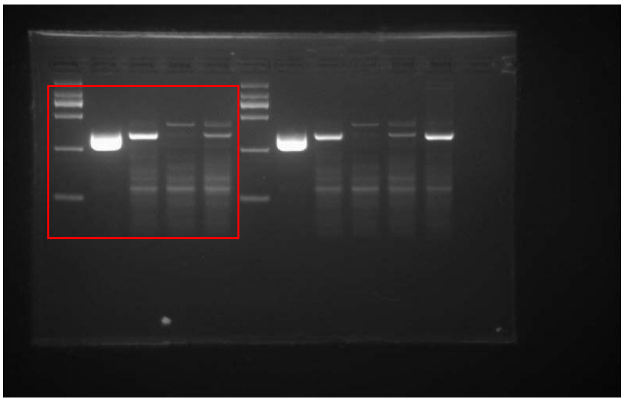

Figure S5 **Full-length gels which have been cropped in the main text are presented.**

A. PCR result in **Fig 1c** of the main text was in the upper pannel, red lines represent cropping lines;T7E1 result in Fig1c was in the down pannel,red lines represent cropping lines. All gels

have been run under the same experimental conditions.

B. **Fig 2c** in the main text was shown here. 5’ Junction PCR(red lines) and 3’ Junction PCR results (green lines)are respectively cropped from the upper pannel, and the Hbb endogenous gene control PCR (1700bp)result (red lines) was cropped from the down pannel. All the gels have been run under the same experimental conditions.

C. **Fig 2d** in the main text was shown here.5’ Junction PCR(red lines) and 3’ Junction PCR results (green lines)are respectively cropped from the upper pannel, and the Hbb endogenous gene control PCR (2830bp)result (red lines) was cropped from the down pannel. All the gels have been run under the same experimental conditions.

D. PCR result in **Fig 3a** of the main text was in the upper pannel, red lines represent cropping lines;T7E1 result in **Fig 3a** was in the down pannel,red lines represent cropping lines. All gels have been run under the same experimental conditions.

E. PCR result in **Fig 3b** of the main text was in the upper pannel, red lines represent cropping lines;T7E1 result in **Fig 3b** was in the down pannel,red lines represent cropping lines. All gels have been run under the same experimental conditions.

F. PCR result in **Fig 3c** of the main text was in the upper pannel, red lines represent cropping lines;T7E1 result in **Fig 3c** was in the down pannel,red lines represent cropping lines. All gels have been run under the same experimental conditions.

G. PCR result in **Fig 3d** of the main text was in the upper pannel, red lines represent cropping lines;T7E1 result in **Fig 3d** was in the down pannel,red lines represent cropping lines. All gels have been run under the same experimental conditions.

H. PCR result in **Fig 5c** of the main text was shown here, the red lines represent the cropping lines.

TABLES

Table 1. The top 10 potential off-target sites of TALENs for the recognition of HBB IVS-2 654 loci.

Serial number	Gene name	TAL 1 Target	TAL 2 Target	Spacer Length
on target	HBB	T ATATGCAGAGATATT	T AACAGTGATAATTTCT	16
Site-1	C4orf21	T AAAAAAAGAAAAATT	T ATATACAAAAATATT	20
Site-2	TTLL7	T AGATGCAAAAATCCT	T ATATCCACAAATATT	25
Site-3	TEX41	T GAAAATCATAATCTCT	T AAATACAGAAATATT	18
Site-4	EGFR	T ATATGTACAAATAAA	T ATATACAAAAATATA	22
Site-5	CTNNA3	T ATATGCCGAAATATT	T AAAACTAATCACTTCT	25
Site-6	SMEK2	T ATATACATAAATATT	T TACAATGCTAATTTAT	25
Site-7	PTPRD	T ATATACAAACATATT	T ATATGCACATATATG	20
Site-8	GPR98	T AACAATAATAATCTCT	T ATATACACACTCATT	26
Site-9	SLC9A6	T ATATGTAGATATATA	T ATATGCAGAAATATG	30
Site-10	TTC28	T ATATGCAGAAATATG	T AATAGATATAACTTCT	25

Table 2. The top 10 potential off-target sites of CRISPR/Cas9 for the recognition of HBB IVS-2 654 loci. Lowercase nucleotides represent a mismatch compared with the on-target sequences.

Serial number	Gene name	Sequence	Orientation	No. of mismatches
on target	HBB	CAGTGATAATTTCTGGGTTAAGG	-	0
Site-1	HHAT	CAGTGATtATTTCTGGGTTATGG	-	1
Site-2	NTRK2	CAGTGATAATTTCaGGGgTATGG	+	2
Site-3	ATXN10	gAGTGATgATTTCTGGGTTAAGG	+	2
Site-4	TPRG1	CAGTGATtATTTCTGGGTggTGG	+	3
Site-5	EHF	tAGTGATAgTTTCTGGGTgAAGG	+	3
Site-6	CCDC178	CtaTGATAATTTCTtGGTTAGGG	+	3
Site-7	CDC7	CAGTGtTAATTTCTGttTTATGG	-	3
Site-8	RNF216	CAGTGActATTTCTGGGTaAGGG	+	3
Site-9	CNTNAP2	gAaTGATAcTTTCTGGGTTAGGG	-	3
Site-10	C2orf73	CAGTGgTAATTTCaGaGTTAAGG	-	3

Table S1

Primer used for PCR and vector construction.

Name	Sequence(5'-3')
HBB-Larm-F	GAACGTGGATGAAGTTGGTGGTGAG
HBB-Larm-R	CCCAGAAATTATCACTGTTATTCTT
HBB-Rarm-F	GGCAATAGCAATATtTCTGCATATAA
HBB-Rarm-R	AGACTGTGAAAGAGTGATAGTTCCG
5' Junction-F	AGAGTTTTCATCCATTCTGTCCTG
5' Junction-R	ACGTGCTACTTCCATTTGTCACGT
3' Junction-F	AATCGCGAACATCTACACCACACAACACCG
3' Junction-R	AGAGAGGACAAGGACCACTTGAGACTCATA
HBB-Endo-F	GTGAGTCTATGGGACGCTTGATGTT
HBB-Endo-R	CTGTGGGAGGAAGATAAGAGGTATG
HBB-T7E1-F	ATCTCTTTCTTTCAGGGCAATAATG
HBB-T7E1-R	TGGGAGGAAGATAAGAGGTATGAAC
Puro△tk-F	GTCACCGAGCTGCAAGAACT
Puro△tk-R	GCCCGAAACAGGGTAAATAA
TALEN-OT1-F	GAGATTACAGACTCCTGCCACTACG
TALEN-OT1-R	ACTCCATCTCAAAAAAAAAACAACA
TALEN-OT2-F	AGGTTTTTTGTACCTGCTCTTTACG
TALEN-OT2-R	CTGAAAAAGAAATGTCCAGACCAAG
TALEN-OT3-F	TGTTTGAAACCAGGGAGAATAAATA
TALEN-OT3-R	ATACTTGCCATAAAAAGGTTTCGTT
TALEN-OT4-F	CTTTGACGATGAAAACATCAAGTTC
TALEN-OT4-R	TACGTATGAACAGTGCCCAGTGTAC
TALEN-OT5-F	AGCTGAGATTAGAGTCCAAAGCTAA
TALEN-OT5-R	GGCTATTTCATCTGTGACAACAAGA
TALEN-OT6-F	AGAATGGTCTCAAACTCCTGACCTC
TALEN-OT6-R	CTGGTCTCTAACTCCTGGGCTCAAC
TALEN-OT7-F	AGATGAAGCTAACATTTGAATTGGC
TALEN-OT7-R	AGTAAAAGACAGAACTCTGGTGGTG
TALEN-OT8-F	GAATGTAATTCTTGTGAATGGCAAA
TALEN-OT8-R	ATCTAAAACCCTCATCTGTCAAGCT
TALEN-OT9-F	ACTCCCTGGAACTATAAGATGCTTC
TALEN-OT9-R	ATGGGAACACTGGTTAACAATTCTG
TALEN-OT10-F	CCTGACTGAAGTGGATTTTTTGTTT
TALEN-OT10-R	TCTGGATGAGTGCGTGATCTTAGAT
CRISPR/Cas9-OT1-F	GCTGAGAGATAAAAGAGGAAAAAGA
CRISPR/Cas9-OT1-R	TTTTACTCAAAATTGGAATAGCTGG
CRISPR/Cas9-OT2-F	TCATTTATGGATTATCTATGGCTGC
CRISPR/Cas9-OT2-R	TTTGTACACATAAGGTTTGGACACC

CRISPR/Cas9-OT3-F	GTTGCAGGGAAGAAGATAATTGACT
CRISPR/Cas9-OT3-R	AGACTATCCTTCATTAAATGATCCT
CRISPR/Cas9-OT4-F	GTATTGTTATGCTTTTAACCCCACG
CRISPR/Cas9-OT4-R	ATGCCAGGATCCTTCTGTTATTTCT
CRISPR/Cas9-OT5-F	AACTAACTCCCTGACACTTAAGGCC
CRISPR/Cas9-OT5-R	CTTTCAACCATTTCCCACACTGTAC
CRISPR/Cas9-OT6-F	AGTTTATGTTGTGTGGGATTTATGC
CRISPR/Cas9-OT6-R	AACAAGGGAATTAAATTTTAGTGGC
CRISPR/Cas9-OT7-F	TCAAGTGATCCCCTCCTATCTTGGC
CRISPR/Cas9-OT7-R	GTTTCCTCTGTTTGGAATGCTCTTC
CRISPR/Cas9-OT8-F	AAATGAAAATAAGTGTGATACCATT
CRISPR/Cas9-OT8-R	CTATCCCCAGAGTTCAGTAGTTCTG
CRISPR/Cas9-OT9-F	TTATTATTGAAGGCCCAAAGTAACT
CRISPR/Cas9-OT9-R	TAGTGGCACACGCCTATAATCCTAC
CRISPR/Cas9-OT10-F	TCTGTTGTCTGAGGATTTTTCAGAT
CRISPR/Cas9-OT10-R	TGGTGGACTCTAAGATTAAAGACCT
HBBcDNA-F	CCTGAGGAGAAGTCTGCCGTTACTGCC
HBBcDNA-R	GCATTAGCCACACCAGCCACCACTT
qHBB-F	TCTGTCCACTCCTGATGCTGTTATG
qHBB-R	GTTGGACTTAGGGAACAAAGGAACC
